# Involvement of mitochondrial ATP-sensitive K^+ ^channels in fentanyl-induced mitochondrial dysfunction of cultured human hepatocytes

**DOI:** 10.1186/cc12325

**Published:** 2013-03-19

**Authors:** S Djafarzadeh, M Vuda, J Takala, SM Jakob

**Affiliations:** 1 Bern University Hospital (Inselspital) and University of Bern, Switzerland

## Introduction

Pharmacological agents used to treat critically ill patients may alter mitochondrial function. The aim of the present study was to investigate whether fentanyl, a commonly used analgesic drug, interacts with hepatic mitochondrial function.

## Methods

The human hepatoma cell line HepG2 was exposed to fentanyl at 0.5, 2 or 10 ng/ml for 1 hour, or pretreated with naloxone (an opioid receptor antagonist) at 200 ng/ml or 5-hydroxydecanoate (5-HD; a specific inhibitor of mitochondrial ATP-sensitive K^+ ^(KATP) channels) at 50 μM for 30 minutes, followed by incubation with fentanyl at 2 ng/ml for an additional hour. The mitochondrial complex I-dependent, II-dependent and IV-dependent oxygen consumption rates of the permeabilized cells were measured using a high-resolution oxygraph (Oxygraph-2k; Oroboros Instruments, Innsbruck, Austria). The respiratory electron transfer capacity of intact cells was evaluated using FCCP (carbonyl cyanide *p*-trifluoromethoxyphenylhydrazone) to obtain the maximum flux.

## Results

**Figure 1:**
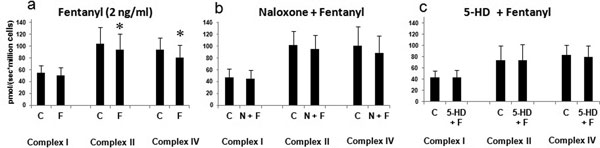
**Cellular oxygen consumption after incubation with fentanyl, naloxone or 5-HD**.

Incubation of HepG2 cells with fentanyl (1 hour, 2 ng/ml) induced a reduction in complex II-dependent and IV-dependent respiration (Figure 1). Cells pretreated with 5-HD before the addition of fentanyl exhibited no significant changes in complex activities in comparison with controls. Pretreatment with naloxone tended to abolish the fentanyl-induced mitochondrial dysfunction. Treatment with fentanyl led to a reduction in cellular ATP content (0.24 ± 0.06 in controls vs. 0.17 ± 0.14 μmol/mg cellular protein in stimulated cells; *P *= 0.02). We did not observe any difference in basal or FCCP-uncoupled respiration rates of cells treated with fentanyl at 2 ng/ml compared with controls (data not shown).

## Conclusion

Fentanyl reduces cultured human hepatocyte mitochondrial respiration by a mechanism that is blocked by a KATP channel antagonist. In contrast, antagonism with naloxone does not seem to completely abolish the effect of fentanyl.

